# Diagnosis and Pathogenesis of Sarcopenia in Chronic Liver Disease Using Liver Magnetic Resonance Imaging

**DOI:** 10.7759/cureus.24676

**Published:** 2022-05-02

**Authors:** Atsushi Nakamura, Tsubasa Yoshimura, Tomomi Sato, Takeshi Ichikawa

**Affiliations:** 1 Hepatology, Nippon Koukan Hospital, Kawasaki, JPN; 2 Gastroenterology, Nippon Koukan Hospital, Kawasaki, JPN

**Keywords:** liver cirrhosis, proton density fat fraction, adipopenia, sarcopenia, magnetic resonance elastography

## Abstract

Background

Liver magnetic resonance imaging (MRI) is rarely used to evaluate sarcopenia. This study sought to develop new diagnostic criteria for MRI in Asians and investigate the relationship between adipopenia and sarcopenia using MRI proton density fat fraction (PDFF), which is correlated with body fat mass.

Methodology

This study included 512 patients with chronic liver disease (CLD) who underwent magnetic resonance elastography (MRE). The following parameters were assessed: paraspinal muscle area/height index (PSMI) measured at the level of the superior mesenteric artery and PDFF. The cutoff PSMI and PDFF values for the diagnosis of sarcopenia and adipopenia, respectively, were determined using receiver operating characteristic analysis of Asians with low body mass index.

Results

Among patients with CLD, the prevalence rates of sarcopenia and adipopenia were 25% and 17%, respectively. We found that sarcopenia increased from stage 3 fibrosis and was inversely correlated with steatosis grade. Multivariate analysis found that MRI-PDFF was associated with sarcopenia. The Kaplan-Meier method in cirrhosis (n = 122) showed that the non-sarcopenia, sarcopenia, and sarcopenia/adipopenia groups had three-year survival rates of 97%, 55% (p < 0.01), and 23%, respectively. The Cox proportional hazards model identified the Child-Pugh score and sarcopenia/adipopenia as independent prognostic factors.

Conclusions

The new diagnostic criteria for sarcopenia confirmed that the prognosis of cirrhosis can be stratified. Furthermore, sarcopenia with adipopenia was shown to be a phenotype of severe sarcopenia in cirrhosis, and screening for sarcopenia should include cases in the precirrhotic stage.

## Introduction

Sarcopenia has been recognized as one of the most important complications of cirrhosis given evidence of its association with a worse prognosis [1]. However, no effective treatment has been established for sarcopenia in patients with cirrhosis. Therefore, the European Association for the Study of the Liver (EASL) and the European Society for Clinical Nutrition and Metabolism (ESPEN) guidelines have recommended screening for sarcopenia with emphasis on early detection [2,3]. The muscle area measured at the third lumbar vertebra (L3) on computed tomography (CT) is often used to determine skeletal muscle mass in chronic liver disease (CLD) and has been reported to be significantly associated with the survival of patients with cirrhosis [4,5]. Therefore, the latest 2021 American Association for the Study of Liver Diseases (AASLD) practice guidance defined the loss of muscle mass as a phenotype of sarcopenia [6]. However, racial differences need to be considered when assessing skeletal muscle mass given that the Global Leadership Initiative on Malnutrition (GLIM) criteria [7], the international diagnostic criteria for malnutrition, set reference values for body mass index (BMI) according to race and age.

Liver magnetic resonance imaging (MRI) has been a widely used imaging modality for the diagnosis of CLD. Notably, contrast-enhanced MRI has been reported to have high detection rates of hepatocellular carcinoma (HCC) [8]. Recently, magnetic resonance elastography (MRE) and proton density fat fraction (PDFF), which are measured using MRI, have been used to diagnose liver fibrosis stage and fatty liver [9,10]. Nevertheless, MRI is rarely used in clinical practice to diagnose sarcopenia. MRI has the great advantage of no radiation exposure, and repeat MRI scans are possible. Moreover, it is also suitable for assessing body composition because of the clear contrast between adipose and muscle tissues. However, considering that liver MRI is rarely performed up to the L3 level, a new anatomical landmark is needed to measure muscle mass. Praktiknjo et al. [11] measured paraspinal muscle area (PSMA) at the level of the origin of the superior mesenteric artery (SMA) and were the first to show that loss of muscle mass is associated with a worse prognosis in patients with decompensated cirrhosis.

Adipopenia has also been associated with a worse prognosis among patients with cirrhosis [12,13]. Fatty acids are the largest stored energy source in the human body, whereas skeletal muscle is one of the tissues that consume the most energy. Additionally, the liver, a central hub of lipid metabolism, receives and stores fatty acids from adipose tissue and distributes lipids as energy substrates to other organs, such as skeletal muscle, according to the energy demand of the body [14,15]. Therefore, we hypothesized that a decrease in hepatic fat may be associated with sarcopenia in patients with cirrhosis. Because MRI-PDFF can accurately measure hepatic fat content [10] and hepatic PDFF has also been shown to strongly correlate with visceral fat and subcutaneous fat area (SFA) [16,17], we focused on PDFF in the evaluation of adipopenia.

The aims of this study were two-fold. First, we sought to develop a new set of diagnostic criteria for sarcopenia based on MRI that is suitable for Japanese and other Asian populations and examine its association with worse prognosis in patients with cirrhosis. Second, we sought to analyze the relationship between adipopenia and the pathogenesis of sarcopenia by measuring PDFF.

## Materials and methods

Study population

This retrospective, single-center study included 512 of 609 patients who underwent MRE between April 01, 2016, and July 31, 2021. The purpose of MRE was to diagnose liver fibrosis and steatosis, as well as the presence of liver tumors on non-contrast MRI images. Overall, 97 patients were excluded due to missing height or weight records (76 patients), steroid or hormone therapy (eight patients), and unmeasurable muscle mass owing to poor image quality or metal artifacts from spinal fusion surgery (13 patients). The CLD etiologies included hepatitis B, hepatitis C, nonalcoholic fatty liver disease (NAFLD), alcoholic fatty liver disease (ALD), and others in 105, 137, 154, 55, and 61 patients, respectively. All patients with hepatitis C who achieved sustained virological response (SVR) with treatment were included, and cirrhosis was diagnosed based on histological or clinical findings, including liver imaging findings. Clinical findings included typical ultrasound findings, low platelet count (<100,000/μL), complications (e.g., varices), and liver imaging findings [1]. ALD was diagnosed according to the diagnostic criteria of the Japanese Society for Biomedical Research on Alcohol [18], whereas NAFLD was diagnosed based on imaging findings (hepatic and renal contrast on abdominal ultrasonography, liver/spleen ratio <0.9 on abdominal CT, and MRI-PDFF >5.2% [19]) and nondrinkers (pure ethanol equivalent, <30 and <20 g/day for males and females, respectively).

This study was approved by the Ethical Review Committee of the Nippon Koukan Hospital (approval number: 202014) and was conducted in compliance with the Ethical Principles for Medical Research Involving Human Subjects described in the Declaration of Helsinki in 1975 (revised in 2000). Informed consent was obtained from the participants via the opt-out approach.

Data collection

Age, sex, height, weight, BMI, and baseline laboratory data were collected from each patient. Cirrhosis severity was assessed using the Child-Pugh score and MELD Na score [20], with disease progression being classified into Child-Pugh classes A, B, and C. The mean and median intervals between clinical data and MRI dates used in the analysis were 9 ± 10 days and 7 (0-16) days, respectively.

MRI protocol

All patients fasted overnight (>12 hours) before evaluation using a 1.5-T whole-body MRI system (SIGNA Voyager XT 1.5T; GE Healthcare, Tokyo, Japan). MRI was performed using the standard protocol, which included T1- and T2-weighted imaging (WI) with fat suppression in axial and coronal views and diffusion-weighted imaging. Liver stiffness measurement (LSM) was measured via elastography to determine hepatic fibrosis progression [21], whereas intrahepatic fat content was measured using IDEAL IQ as PDFF [22]. The respective measurements of LSM (kPa) and PDFF (%) were analyzed by radiologists. The method of liver elastography was outlined as follows: a 19 cm diameter passive pneumatic driver connected to an acoustic waveform generator was placed over the center of the patient’s right thorax at the level of the xiphoid process, and the propagated 60 Hz acoustic waves were collected and analyzed in the gradient-echo sequence of the MRI image. The region of interest was carefully placed in the right liver lobe, avoiding blood vessels, bile ducts, gallbladder, tumors, and artifacts. The diagnosis of hepatic fibrosis stage was made according to two criteria for each etiology, considering that liver stiffness measured by MRE differed depending on the etiology. NAFLD was diagnosed using the criteria of Hsu et al. [23] (fibrosis stage 0, fibrosis stage 1, fibrosis stage 2, fibrosis stage 3, and fibrosis stage 4, with thresholds of 2.61 kPa, 2.97 kPa, 3.62 kPa, and 4.69 kPa, respectively). The other causes were diagnosed using Morisaka et al.’s [24] criteria (threshold values of 2.32 kPa, 2.61 kPa, 3.02 kPa, and 4.23 kPa, respectively).

**Figure 1:**
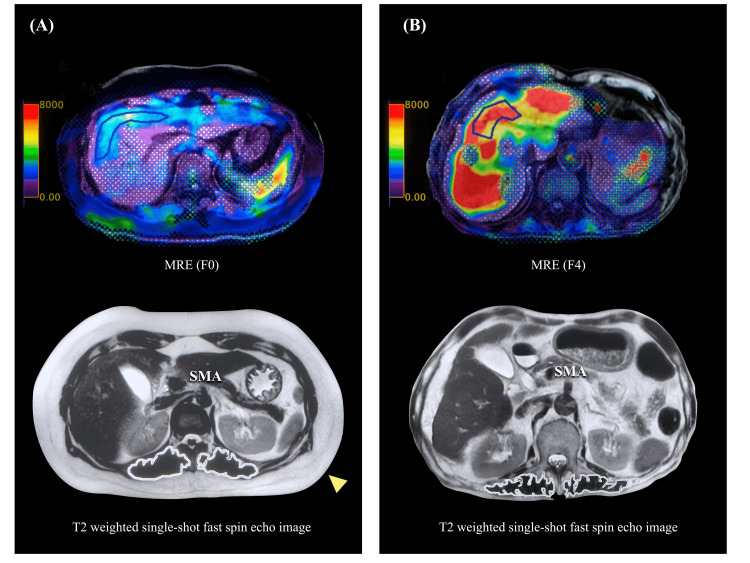
Magnetic resonance imaging. (A) Female case of CLD (F0): the upper panel shows an MRE image, and the lower panel shows a T2-weighted single-shot fast spin-echo image of the origin of the superior mesenteric artery. The white contours correspond to the left and right paraspinal muscle areas (iliopsoas, dorsalis longus, spinal cord, multifidus, and quadratus lumborum). Subcutaneous fat area (yellow arrows) was also measured in this study. (B) Male case in cirrhotic stage (F4); PSMI and fat mass decreased and diagnosed as sarcopenia and adipopenia. CLD: chronic liver disease; MRE: magnetic resonance elastography

MRI image analysis was performed on a separate workstation using a T2-WI single-shot first spin-echo (SS-FSE) sequence (Figure 1).

The sequence parameters for SS-FSE were as follows: echo time, 90 ms; repetition time, 565-644 ms; flip angle, 90; slice thickness, 6 mm; and voxel size, 0.9 × 1.6 × 6.0 mm^3^. The bilateral paraspinal musculature was manually segmented at the level of the origin of the SMA using OsiriX version 6.0 open-source software (64-bit, Pixmeo, Geneva, Switzerland; http://www.osirix-viewer.com). For the PSMA (cm^2^) measurements, two researchers (TY and AN) independently measured the MRI images of 30 randomly selected patients from the same group to assess agreement. Consequently, the correlation coefficient between measurements was 0.94, and one researcher (AN) completed measurements of a series of MRI images. Each measured PSMA was normalized using the square of the patient’s height in meters (m) obtained from electronic medical records to calculate the paraspinal muscle index (PSMI: cm^2^/m^2^). Additionally, this study measured SFA (cm^2^) at the level of the SMA origin.

Diagnosis of sarcopenia and adipopenia

Representatives of the world’s major nutritional societies proposed the GLIM criteria as a new set of diagnostic criteria for malnutrition. This set of criteria requires that younger patients (under 70 years old) be analyzed separately from older patients (over 70 years old). The GLIM criteria defined low BMI in the Asian population as <18.5 and <20.0 kg/m^2^ in the younger and older age groups, respectively. This study used receiver operating characteristic (ROC) analysis of PSMI and PDFF for low BMI to determine the cutoff values for diagnosing sarcopenia and adipopenia, respectively.

Statistical analysis

JMP statistical software (version 12.2; SAS Institute Japan, Tokyo, Japan) was used for all statistical analyses. The chi-square test, Wilcoxon-Mann-Whitney test, and Spearman’s rank correlation coefficient were used for between-group analyses, whereas logistic regression analysis was used to examine factors associated with sarcopenia in CLD. The stepwise increase/decrease method was used to select variables. Patient prognosis was analyzed using the Kaplan-Meier and Cox proportional hazards methods, whereas the stepwise increase/decrease method was used for variable selection. Statistical significance was set at p-values of <0.05.

## Results

Baseline characteristics

The characteristics of all patients are summarized in Table 1.

**Table 1:** Clinical characteristics. Data are presented as mean ± standard deviation or n (%) ALD: alcoholic liver disease; ALP: alkaline phosphatase; ALT: alanine aminotransferase; AST: aspartate aminotransferase; BMI: body mass index; BUN: blood urea nitrogen; γ-GTP: gamma-glutamyl transpeptidase; HCC: hepatocellular carcinoma; MELD Na: Model for End-Stage Liver Disease Sodium; NAFLD: non-alcoholic fatty liver disease; NH_3_: ammonia

	All cases (512)	Noncirrhosis (390)	Cirrhosis (122)	P-value
Age (years)	62 ± 14	61 ± 15	68 ± 12	<0.001
Sex (male/female)	307/205	232/158	75/47	0.816
BMI (kg/m^2^)	25 ± 4	24 ± 4	25 ± 5	0.201
Low BMI	39 (8)	28 (7)	11 (9)	0.481
Etiology of liver disease				
Hepatitis B	105 (21)	91 (23)	14 (11)	
Hepatitis C	137 (27)	100 (26)	37 (30)	
NAFLD	154 (30)	129 (33)	25 (33)	
ALD	55 (10)	22 (6)	22 (6)	
Others	61 (12)	48 (12)	13 (11)	
Clinical data				
Leucocytes (/mm^3^)	5690 ± 1709	5797 ± 1463	5356 ± 2067	0.001
Hemoglobin (g/dL)	13.9 ± 1.9	14.1 ± 1.6	13.1 ± 2.4	<0.001
Platelets (×10^4^/mm^3^)	19.6 ± 6.7	21.3 ± 6.0	13.6 ± 6.0	<0.001
Total bilirubin (mg/dL)	1.0 ± 0.9	0.9 ± 0.4	1.6 ± 1.7	<0.001
AST (IU/L)	39 ± 37	35 ± 32	53 ± 47	<0.001
ALT (IU/L)	40 ± 45	41 ± 49	37 ± 32	0.593
ALP (IU/L)	283 ± 170	243 ± 86	412 ± 241	<0.001
γ-GTP (IU/L)	86 ± 152	73 ± 145	130 ± 160	<0.001
Albumin (g/dL)	4.1 ± 0.5	4.3 ± 0.3	3.6 ± 0.7	<0.001
Total cholesterol (mg/dL)	192 ± 42	202 ± 36	163 ± 43	<0.001
Trigliceride (mg/dL)	146 ± 111	154 ± 116	120 ± 88	<0.001
BUN (mg/dL)	16 ± 9	15 ± 6	17 ± 13	0.579
Creatinine (mg/dL)	0.9 ± 0.8	0.8 ± 0.3	1.1 ± 1.5	0.328
Prothrombin activity (%)	97 ± 16	102 ± 13	83 ± 17	<0.001
NH_3 _(μg/dL)	37 ± 28	28 ± 13	61 ± 40	<0.001
HCC	48 (9)	14 (4)	34 (28)	<0.001
Child-Pugh class (A/B/C)	-	-	68/36/18	-
Child–Pugh score	-	-	7 ± 2	-
MELD Na score	-	-	7 ± 7	-

The median age of all patients was 65 years (range: 15-94). The overall median BMI was 24 kg/m^2^, and low BMI was observed in 39 patients (8%), although no significant difference in BMI was observed between cirrhosis and noncirrhosis cases. Comparing the clinical findings according to etiology showed that the NAFLD group had more young patients (59 ± 16 and 64 ± 13 years old) than the non-NAFLD group (p < 0.01), and the ALD group had a higher proportion of patients with cirrhosis than the non-ALD group (p < 0.01). The prevalence of HCC was the highest in the hepatitis C group (p < 0.01).

Cutoff values for sarcopenia and adipopenia according to MRI image analysis

The mean PSMI was 14.82 ± 3.63 cm^2^/m^2^ and 12.24 ± 3.17 cm^2^/m^2^ for males and females, respectively. The SFAs of males and females were 88.4 ± 41.3 cm^2^ and 121.9 ± 66.4 cm^2^, respectively, suggesting sex differences (p < 0.01). Conversely, PDFF was 8.2% ± 7.5% and 7.7% ± 7.6% in males and females, respectively, with no significant difference (p = 0.170).

**Figure 2:**
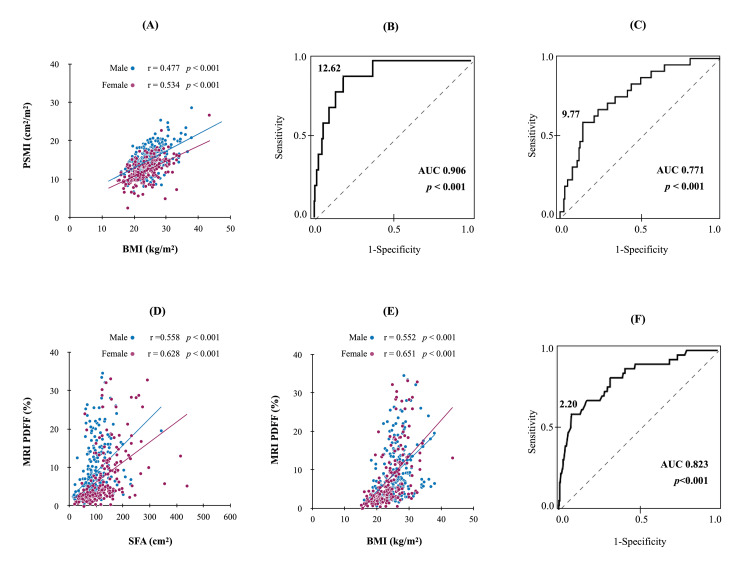
Analysis of the respective cutoff values diagnosing sarcopenia and adipopenia. (A) Correlation between PSMI and BMI according to sex in CLD without ascites. (B) The ROC curve of the PSMI value predicting low BMI in males. The cutoff value was set at 12.62 cm^2^/m^2^. (C) The ROC curve of the PSMI value predicting low BMI in females. A cutoff value of 9.77 cm^2^/m^2^ was set. (D) Correlation between PDFF and subcutaneous fat area in males and females. (E) Correlation between PDFF and BMI. (F) The ROC curve of PDFF predicting low BMI. The cutoff value of PDFF for predicting low BMI was 2.2%. AUC: area under the curve; BMI: body mass index; CLD: chronic liver disease; PDFF: proton density fat fraction; PSMI: paraspinal muscle index; ROC: receiver operating characteristic

Among 477 patients without ascites, PSMI was significantly correlated with BMI in both males and females (Figure 2A). After PSMI was analyzed using the ROC for sex to determine the diagnostic performance of low BMI (Figures 2B, 2C), our results showed an area under the curve (AUC) of 0.906 for male patients (p < 0.001) and 0.771 for female patients (p < 0.001). Thereafter, we determined the cutoff values for males and females and found that a PSMI of <12.62 and <9.77 cm^2^/m^2^ for males and females were optimal for the diagnosis of sarcopenia, respectively. In contrast, liver PDFF was also significantly correlated with BMI (p < 0.001), and a significant positive correlation with SFA was confirmed (p < 0.001). The optimal cutoff value of PDFF for adipopenia diagnosis was 2.2% based on ROC analysis (p < 0.001) (Figures 2D-2F).

Sarcopenia in patients with CLD

Table 2 compares the clinical findings between patients with and without sarcopenia.

**Table 2:** Comparison of clinical findings between patients with and without sarcopenia in those with chronic liver disease. Data are presented as mean ± standard deviation or n (%). ALP: alkaline phosphatase; ALT: alanine aminotransferase; AST: aspartate aminotransferase; BMI: body mass index; BUN: blood urea nitrogen; γ-GTP: gamma-glutamyl transpeptidase; HCC: hepatocellular carcinoma; LSM: liver stiffness measurements; NH_3_: ammonia; PDFF: proton density fat fraction; PSMI: paraspinal muscle index; SFA: subcutaneous fat area

	No sarcopenia	Sarcopenia	P-value
n (%)	385 (75)	127 (25)	
Age (year)	60 ± 14	70 ± 13	<0.001
Sex (male/female)	227/158	80/47	0.167
BMI (kg/m^2^)	25 ± 4	23 ± 5	<0.001
Low BMI	12 (3)	27 (21)	<0.001
PSMI (male/female: cm^2^/m^2^)	16.4 ± 2.7/13.5 ± 2.5	10.3 ± 1.7/8.3 ± 1.5	<0.001
SFA (male/female: cm^2^/m^2^)	96.3 ± 39.9/131.2 ± 61.3	66.7 ± 37.7/94.3 ± 72.4	<0.001
MRI-PDFF (%)	9.2 ± 7.9	4.4 ± 4.7	<0.001
Adipopenia (PDFF ≦ 2.2%)	36 (9)	41 (32)	<0.001
LSM (kPa)	3.23 ± 1.96	4.44 ± 2.66	<0.001
Cirrhosis	64 (17)	58 (46)	<0.001
Ascites	6 (2)	29 (23)	<0.001
HCC	25 (7)	23 (18)	<0.001
Total bilirubin (mg/dL)	1.0 ± 0.8	1.3 ± 1.2	0.019
AST (IU/L)	38 ± 37	41 ± 36	0.102
ALT (IU/L)	44 ± 51	27 ± 17	0.004
ALP (IU/L)	263 ± 134	339 ± 240	<0.001
γ-GTP (IU/L)	79 ± 127	108 ± 209	0.751
Albumin (g/dL)	4.2 ± 0.4	3.7 ± 0.7	<0.001
Total cholesterol (mg/dL)	199 ± 37	171 ± 43	<0.001
Triglyceride (mg/dL)	154 ± 116	119 ± 88	<0.001
BUN (mg/dL)	15 ± 6	18 ± 14	0.006
Creatinine (mg/dL)	0.8 ± 0.3	1.1 ± 1.4	0.599
Leucocytes (/mm^3^)	5838 ± 1506	5235 ± 2162	<0.001
Hemoglobin (g/dL)	14.2 ± 1.8	12.9 ± 2.0	<0.001
Platelets (×10^4^/mm^3^)	20.7 ± 6.2	16.3 ± 7.0	<0.001
Prothrombin activity (%)	99 ± 15	90 ± 18	<0.001
NH_3_ (μg/dL)	34 ± 24	46 ± 37	0.004

**Figure 3:**
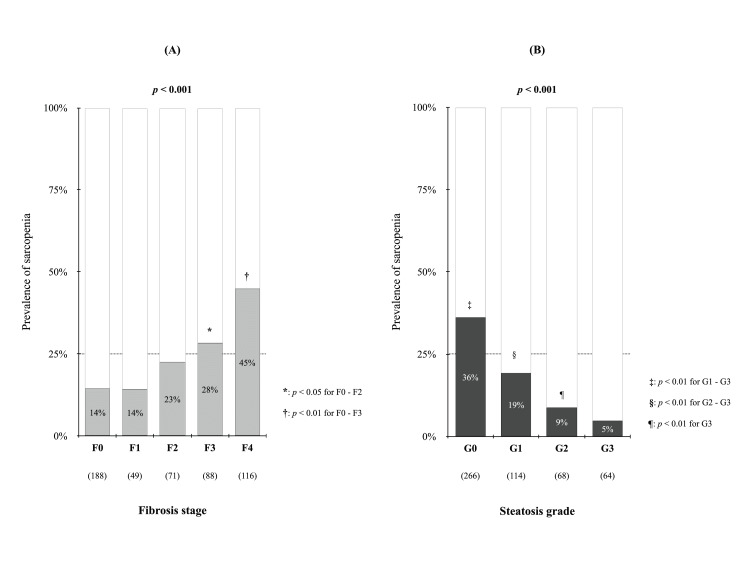
Relationship of sarcopenia to liver fibrosis stage and steatosis grade. (A) Prevalence of sarcopenia according to liver fibrosis stage assessed using MRE. The prevalence of sarcopenia was significantly higher in patients with fibrosis stages 3 and 4 than in those with fibrosis stages 0–2 (p < 0.05). (B) The prevalence of sarcopenia for each steatosis grade based on PDFF measured using MRI. A negative correlation was observed between the prevalence of sarcopenia and the degree of hepatic steatosis (p < 0.001). MRE: magnetic resonance elastography; MRI: magnetic resonance imaging; PDFF: proton density fat fraction

Notably, the sarcopenia group was older, had a higher frequency of low BMI, and had more cases of cirrhosis and HCC than the nonsarcopenia group (p < 0.001 for each). Blood chemistry data showed that the sarcopenia group had lower levels of alanine aminotransferase (ALT), albumin, total cholesterol, triglycerides, urea nitrogen, and prothrombin activity and significantly higher levels of bilirubin, alkaline phosphatase (ALP), and blood ammonia than the nonsarcopenia group (p < 0.05 for each). In the peripheral blood, three types of blood cells were decreased: white blood cells, hemoglobin, and platelets (p < 0.01 for each). Figures 3A, 3B show the prevalence of sarcopenia in each of the liver fibrosis stages according to MRE [23,24] and steatosis grades according to PDFF [19]. In patients with CLD, the prevalence of sarcopenia significantly increased from fibrosis stage 3 (F0-2 vs. F3, p < 0.05) and showed a negative relationship with the degree of hepatic steatosis (p < 0.001).

Multivariate analysis of risk factors associated with sarcopenia in patients with CLD

Multivariate analysis was performed on the following eight factors selected using stepwise logistic regression of factors significantly associated with sarcopenia in univariate analysis: age, low BMI, PDFF, ALT, albumin, total cholesterol, prothrombin activity, and HCC (Table 3). Accordingly, our results showed that age, low BMI, PDFF, albumin, and total cholesterol significantly contributed to sarcopenia in patients with CLD (p < 0.05 for all).

**Table 3:** Factors associated with sarcopenia in chronic liver disease. ALT: alanine aminotransferase; BMI: body mass index; HCC: hepatocellular carcinoma; PDFF: proton density fat fraction

	Univariate analysis		Multivariate analysis	
	Odds ratio (95% CI)	P-value	Odds ratio (95% CI)	P-value
Age (year)	1.059 (1.041–1.079)	<0.001	1.030 (1.007–1.055)	0.009
Low BMI	7.329 (3.526–16.75)	<0.001	5.412 (2.251–13.74)	<0.001
MRI-PDFF (%)	0.837 (0.787–0.888)	<0.001	0.942 (0.887–0.994)	0.029
ALT (IU/L)	0.983 (0.973–0.992)	<0.001		
Albumin (g/dL)	0.144 (0.088–0.229)	<0.001	0.214 (0.104–0.418)	<0.001
Total cholesterol (mg/dL)	0.981 (0.975–0.987)	<0.001	0.991 (0.982–0.999)	0.036
Prothrombin activity (%)	0.963 (0.949–0.975)	<0.001		
Presence of HCC	3.354 (1.811–6.207)	<0.001		

Impact of sarcopenia on the pathogenesis and prognosis of cirrhosis

**Figure 4:**
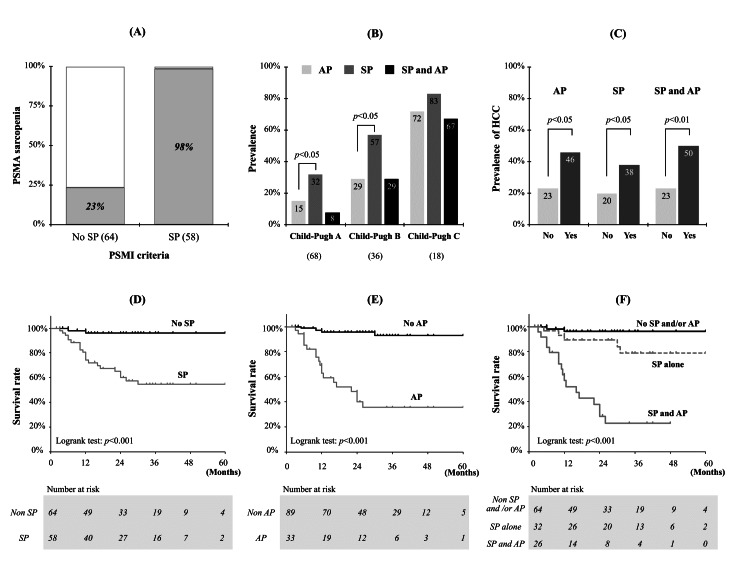
Study of sarcopenia in cirrhosis. (A) Comparison of the prevalence of sarcopenia diagnosed according to the PSMI criteria (male <12.62 cm^2^/m^2^, female <9.77 cm^2^/m^2^) and PSMA criteria (male <3,523 mm^2^, female <3,153 mm^2^) in 122 patients with cirrhosis. Sarcopenia according to the PSMI criteria was found in 58 (48%) patients, 57 of whom also had sarcopenia according to the PSMA criteria. On the other hand, of the 64 patients diagnosed with nonsarcopenia by PSMI criteria, 15 (23%) were diagnosed with sarcopenia by the PSMI criteria. (B) Prevalence of adipopenia and sarcopenia according to the Child-Pugh classification (A, B, and C). In Child-Pugh A and B, sarcopenia was significantly more common than adipopenia. Moreover, the combined type increased with progression. (C) The prevalence of hepatocellular carcinoma in adipopenia, sarcopenia, and combined types. Significant differences were observed in all groups. (D, E) Kaplan-Meier survival curves in cirrhosis patients with and without sarcopenia and with and without adipopenia. (F) Kaplan-Meier survival curves comparing three groups of patients with cirrhosis (no sarcopenia, sarcopenia alone, and sarcopenia plus adipopenia). AP: adipopenia; PSMA: paraspinal muscle area; PSMI: paraspinal muscle index; SP: sarcopenia

Among the 122 patients with cirrhosis, 58 (48%) had sarcopenia. In contrast, 72 (59%) patients had sarcopenia after applying the PSMA criteria (males <3,523 mm^2^, females <3,153 mm^2^) [10]. The Cohen kappa value for both criteria for sarcopenia diagnosis was 0.87, and 21% of sarcopenic patients diagnosed using the PSMA criteria were false-positive according to the PSMI criteria (Figure 4A). The prevalence of sarcopenia according to the Child-Pugh classification (A, B, and C) was 32%, 57%, and 83%, respectively (Figure 4B). Furthermore, adipopenia was found in 27% of the patients. Sarcopenia increased before adipopenia in Child-Pugh A and B patients, with no difference between them in Child-Pugh C patients. In HCC patients (Figure 4C), sarcopenia and adipopenia were significantly more frequent (p < 0.05).

During the mean observation period of 25.0 ± 16.2 months, 24 liver-related deaths occurred, among which 14 were due to liver failure, and 10 were due to HCC. Kaplan-Meier survival curves for cirrhosis (Figures 4D, 4E) showed significant differences in survival rates between patients with and without sarcopenia and adipopenia (p < 0.01). Figure 4F shows the Kaplan-Meier survival curves for cirrhosis divided into three groups (no sarcopenia, sarcopenia alone, and sarcopenia combined with adipopenia). The survival of patients with sarcopenia and cirrhosis was significantly stratified according to the presence of adipopenia complications (hazards ratio: 6.65, 95% confidence interval: 2.59-20.44, p < 0.001).

Univariate analysis based on the Cox proportional hazards model (Table 4) was performed for age; sex; BMI; specific blood parameters; LSM; neutrophil-lymphocyte ratio (NLR); MELD-Na score; Child-Pugh score; and presence of HCC, sarcopenia, and adipopenia. Multivariate analysis with four significant factors selected using the stepwise increase/decrease method showed that Child-Pugh score and sarcopenia combined with adipopenia were independent prognostic factors for cirrhosis (p < 0.001 for both).

**Table 4:** Prognostic factors according to Cox proportional hazards model in cirrhosis. CI: confidence interval; HCC: hepatocellular carcinoma; HR: hazards ratio; MELD Na: Model for End-Stage Liver Disease Sodium; NLR: neutrophil-to-lymphocyte ratio; PSMA: paraspinal muscle area; PSMI: paraspinal muscle index

	Univariate analysis		Multivariate analysis	
	HR (95%CI)	P-value	HR (95% CI)	P-value
Total bilirubin (mg/dL)	1.254 (1.041–1.445)	0.021		
Total cholesterol (mg/dL)	0.981 (0.969–0.992)	0.001		
Albumin (g/dL)	0.239 (0.136–0.419)	<0.001		
Hemoglobin (g/dL)	0.751 (0.625–0.902)	0.002		
NLR	1.182 (1.001–1.334)	0.048	1.139 (0.872–1.354)	0.233
Child-Pugh score	1.570 (1.348–1.834)	<0.001	1.375 (1.147–1.652)	<0.001
Child-Pugh classification (C/AB)	8.304 (3.685–18.58)	<0.001		
MELD Na	1.136 (1.074–1.445)	<0.001		
Presence of HCC	2.711 (1.169–6.423)	0.021		
Sarcopenia (PSMA)	4.167 (1.595–14.25)	<0.001		
Sarcopenia (PSMI)	8.584 (2.974–38.26)	<0.001	1.470 (0.371–6.131)	0.577
Adipopenia	9.292 (4.030–29.99)	<0.001		
Sarcopenia (PSMI) and adipopenia	11.59 (5.107–28.14)	<0.001	4.768 (1.829–15.04)	<0.001

## Discussion

The current study presented the first diagnostic criteria for sarcopenia based on MRI imaging in Asian populations. From a cohort of CLD patients who underwent MRE, we showed the validity of measuring muscle area using SMA as a landmark for the diagnosis of sarcopenia and found that sarcopenia in CLD increases from the precirrhotic stage. Furthermore, our data revealed that adipopenia also affects the prognosis of cirrhosis.

The assessment of sarcopenia in CLD has been based mainly on muscle cross-sectional area at the L3 level determined using CT [25]. In contrast, MRI has not been widely used to establish a clinical diagnosis of sarcopenia considering that MRI is expensive, nonradiologists have difficulty processing the various images, and previous reports have used fat-free skeletal muscle areas postprocessed with specialized analysis software [11,26,27]. Furthermore, liver MRI rarely includes the L3 level, which is a landmark area for diagnosing sarcopenia using CT. Derstine et al. [28], who measured the skeletal muscle index at each vertebral level from the thoracic to the lumbar spine using CT images from 735 healthy individuals, showed that sarcopenia can be diagnosed at levels other than L3. Moreover, Praktiknjo et al. [11] found that the diagnostic performance of two MRI parameters (PSMA, fat-free PSMA) measured at the level of SMA was equivalent in ROC analysis to predict the three-year survival rates of patients with cirrhosis. Based on the aforementioned results, the current study diagnosed sarcopenia by measuring PSMI using the origin of SMA as a landmark. In addition, T2-weighted SS-FSE used for image analysis has very good visualization of SMA and contrast between skeletal muscle and adipose tissue, which is suitable for measuring muscle cross-sectional area.

**Figure 5:**
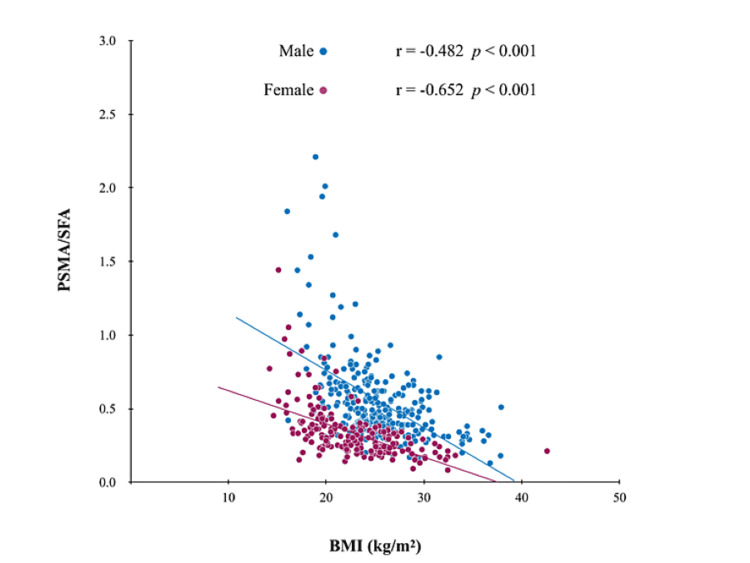
Relationship between BMI and body composition in men and women. Sex differences in the correlation between BMI and PSMA/SFA in CLD. In the present study, BMI was negatively correlated with the ratio of muscle mass to fat mass in both males and females, and the sex difference was more pronounced at lower BMI values. BMI: body mass index; CLD: chronic liver disease; PSMA: paraspinal muscle area; SFA: subcutaneous fat area

Indeed, racial differences in body size and muscle mass have been noted, with studies showing that the average muscle mass of Asians is approximately 15% less than that of Western populations [29]. Therefore, new reference values for the Asian population are needed to diagnose sarcopenia using MRI. In this study, the cutoff value for sarcopenia was determined using PSMI, which corresponds to low BMI in Asians according to the GLIM criteria. The results showed that the cutoff values were 12.62 and 9.77 cm^2^/m^2^ for males and females, respectively. On the other hand, we noticed a difference in AUC between males and females in the ROC analysis. As an additional analysis (Figure 5), we examined the relationship between BMI and the ratio of muscle mass to fat mass (PSMA/SFA). We found a negative correlation between BMI and the muscle-to-fat ratio in both males and females. Furthermore, the sex difference in PSMA/SFA increased with decreasing BMI, and PSMA/SMA was significantly higher in males than in females in the low BMI cases (p < 0.05). Therefore, the sex differences in body composition were more pronounced in the malnourished condition, and this was thought to explain the differences in AUC.

Previous reports have shown that the prevalence of sarcopenia in cirrhotic patients ranges from 30% to 70%, and the annual rate of skeletal muscle mass loss accelerates with liver severity: 1.3% in Child-Pugh A, 3.5% in Child-Pugh B, and 6.1% in Child-Pugh C [30]. The current study, which used the PSMI criteria, revealed a similar prevalence of sarcopenia in cirrhosis (48%) and increased with the progression of the Child-Pugh classification. Moreover, a recent large meta-analysis by Tantai et al. [31], which compared the prognosis of sarcopenia, showed that the one-year and three-year survival rates for sarcopenic patients were 77% and 64%, respectively. In contrast, such rates for our patients were 80% and 55%, respectively, which appears to be consistent with previous reports. However, we also showed that the cutoff value by the GLIM criteria corrected the overdiagnosis in Asians by the PSMA criteria used in previous reports. Although MRI offers a great advantage given the lack of radiation exposure, one of its disadvantages is its long examination time. However, image reconstruction using deep learning methods of artificial intelligence has the potential to improve this problem [32]. Furthermore, noncontrast MR is currently attracting attention as a screening method for HCC [33]. In this study, eight patients were diagnosed with new HCC based on imaging data from the MRE examination. Therefore, we believe that our proposed sarcopenia diagnosis method utilizing liver MRI is practical and will be beneficial for CLD patients in the future.

The strength of this study is that we assessed sarcopenia using imaging data obtained from MRE examinations. This allowed us to analyze the prevalence of sarcopenia according to the hepatic fibrosis stage in CLD, with our findings showing a significant difference in the prevalence of sarcopenia between liver fibrosis stage 0-2 and fibrosis stage 3. The results are important, and the AASLD practice guidance, which covers cirrhosis, suggests routine screening as the primary prevention of sarcopenia and early intervention based on appropriate diagnosis as the secondary prevention, with such interventions aimed at delaying the onset and progression of sarcopenia. The present data showed no difference in the prevalence of sarcopenia between patients with fibrosis stage 3 and those with Child-Pugh A (28% vs. 32%, p = 0.633). Therefore, we believe that patients with advanced liver fibrosis should also be screened for sarcopenia.

Another finding of this MRI study was that, among patients with CLD, decreased PDFF was strongly associated with decreased muscle mass, which appeared consistent with our hypothesis. Notably, among patients with cirrhosis, the poor prognosis of those with sarcopenia was determined by the presence of adipopenia. Previous studies have reported that portal hypertension and PEM are associated with lower body fat mass in cirrhosis [34,35]. A study using indirect calorimetry by Glass et al. [36] reported that energy expenditure was increased in cirrhotic patients and that the decrease in the respiratory quotient, which reflects fat burning, was significantly associated with decreased muscle and body fat mass. Conversely, evidence has shown that correction of energy depletion by nocturnal nutritional supplementation increases total body protein and fat-free mass in cirrhosis [37]. These results predict the existence of crosstalk between muscle and adipose tissue in the regulation of energy metabolism in cirrhosis, with our results appearing to be consistent with these data. In fact, Shimizu et al. [38] showed that amino acid supply to the liver by skeletal muscle catabolism suppressed fibroblast growth factor-21-induced lipolysis in mice, indicating an axis of control of fat mass by skeletal muscle. Moreover, this study showed that the combination of sarcopenia and adipopenia was a poor prognostic factor for cirrhosis independent of liver function. Given that we diagnosed sarcopenia and adipopenia based on the GLIM criteria, these results may reflect the severity of malnutrition in cirrhosis. In other words, sarcopenia with adipopenia can be regarded as a new phenotype of severe sarcopenia. Our results indicate that suppression of adipopenia may be a new therapeutic target for sarcopenia in patients with cirrhosis.

**Figure 6:**
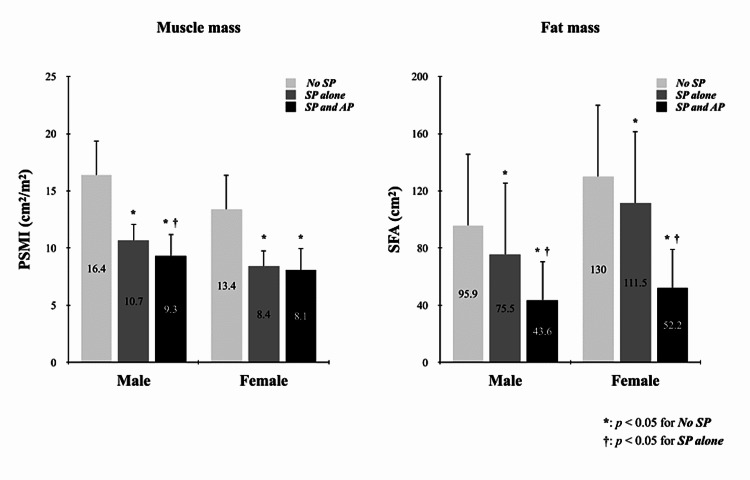
Comparison of muscle mass and fat mass in three groups (no sarcopenia, sarcopenia alone, sarcopenia plus adipopenia) of cirrhotic patients. The patients with sarcopenia and adipopenia had the lowest muscle and fat mass in both sexes. AP: adipopenia; PSMI: paraspinal muscle index; SFA: subcutaneous fat area; SP: sarcopenia

There are several limitations to this study. First, given that this was a single-center retrospective analysis, the cutoff values for Asians need to be validated in other centers. Second, we assessed adipopenia by measuring liver PDFF on MRI. Studies have pointed out that liver fat content is associated with hepatocyte function as well as adipose tissue. Enooku et al. [39] reported that hepatic fat loss in NASH was associated with decreased expression of fatty acid transport protein 5 due to progressive fibrosis. To this end, we compared muscle and fat mass in the no sarcopenia group, the sarcopenia alone group, and the combined sarcopenia and adipopenia group (Figure 6). The results confirmed that sarcopenia with adipopenia resulted in the lowest SFA in both sexes (p < 0.05) and that lower PDFF was associated with lower body fat mass in cirrhosis. Therefore, our study using PDFF as an indicator of adipopenia seems to be acceptable. Third, we set the same PDFF value as the criterion for adipopenia in NAFLD and non-NAFLD patients. Therefore, the criteria for adipopenia in NAFLD patients should be reconsidered.

## Conclusions

Liver MRI is promising and suitable for the diagnosis of sarcopenia. Therefore, we proposed a new set of criteria for the diagnosis of sarcopenia using MRI in Asians. This study suggests that sarcopenia with adipopenia is a phenotype of severe sarcopenia and that screening for sarcopenia is necessary for patients with advanced fibrosis stage.
